# Intramedullary bone tissue reaction of ion-releasing resin-modified glass-ionomer restoration versus two calcium silicate-based cements: an animal study

**DOI:** 10.1038/s41598-023-36949-w

**Published:** 2023-06-17

**Authors:** Ashraf Abou ElReash, Mohamed Grawish, Walied Abdo, Amr M. Abdelghany, Luo Junsi, Xie Xiaoli, Hamdi Hamama

**Affiliations:** 1 Department of Conservative Dentistry. Faculty of Oral and Dental Medicine, Delta University for Science and Technology, Mansoura, Egypt; 2 Department of Oral Biology, Faculty of Dentistry, Mansoura University, Mansoura, Egypt; 3 Department of Oral Biology, Faculty of Oral and Dental Medicine, Delta University for Science and Technology, Mansoura, Egypt; 4 Department of Pathology, Faculty of Veterinary Medicine, Kafr Elsheikh University, Kafr El Sheikh, Egypt; 5 Department of Spectroscopy, Physics Research Institute, National Research Centre, Giza, Egypt; 6 Department of Endodontic. Xiangya School of Stomatology. Central, South University, Changsha, Hunan Province China; 7 Department of Conservative Dentistry, Faculty of Dentistry, Mansoura University, Mansoura, 35516 Egypt; 8 Faculty of Dentistry, New-Mansoura University, New-Mansoura, Egypt

**Keywords:** Dental materials, Endodontics, Restorative dentistry

## Abstract

This comparative study was conducted to assess the intramedullary bone tissue reaction of an ion-releasing resin modified glass-ionomer cement with claimed bioactivity (ACTIVA bioactive resin) restorative material versus Mineral Trioxide Aggregate High Plasticity (MTA HP) and bioceramic putty iRoot BP Plus. Fifty-six adult male Wistar rats were assigned into 4 equal groups (14 rats each). A surgical intramedullary bi-lateral tibial bone defects were performed in rats of the control group I (GI) and left without any treatment to be considered as controls (n = 28). The rats of groups II, III and IV were handled as group I except that the tibial bone defects were filled with ACTIVA, MTA HP and iRoot BP, respectively. In all groups, rats were euthanized after one month and specimens were processed to histological investigation, SEM examination and EDX elemental analysis. In addition, semi-quantitative histomorphometric scoring system was conducted for the following parameters; new bone formation, inflammatory response, angiogenesis, granulation tissue, osteoblasts and osteoclasts. The clinical follow-up outcome of this study revealed the recovery of rats after 4 days post-surgical procedure. It was observed that the animal subjects returned to their routine activities, e.g., walking, grooming and eating. The rats showed normal chewing efficiency without any weight loss or postoperative complications. Histologically, the control group sections showed scanty, very thin, new bone trabeculae of immature woven type located mostly at the peripheral part of the tibial bone defects. These defects exhibited greater amount of thick bands of typically organized granulation tissue with central and peripheral orientation. Meanwhile, bone defects of ACTIVA group showed an empty space surrounded by thick, newly formed, immature woven bone trabeculae. Moreover, bone defects of MTA HP group were partially filled with thick newly formed woven bone trabeculae with wide marrow spaces presented centrally and at the periphery with little amount of mature granulation tissue at the central part. The iRoot BP Plus group section exhibited an observable woven bone formation of normal trabecular structures with narrow marrow spaces presented centrally and at the periphery showed lesser amount of well-organized/mature granulation tissue formation. Kruskal Wallis test revealed total significant differences between the control, ACTIVA, MTAHP and iRoot BP Plus groups (p < 0.05). Meanwhile, Mann–Whitney U test showed significant difference between control and ACTIVA groups, Control and MTA HP groups, control and iRoot BP Plus groups. ACTIVA and MTA HP groups, ACTIVA and iRoot BP Plus (p ˂ 0.05) with no significant difference between MTA HP and iRoot BP Plus (p > 0.05). The elemental analysis outcome showed that the lesions of the control group specimens were filled with recently created trabecular bone with limited marrow spaces. EDX tests (Ca and P analysis) indicated a lower degree of mineralization. Lower amounts of Ca and P was expressed in the mapping analysis compared with other test groups. Calcium silicate-based cements induce more bone formation when compared to an ion-releasing resin modified glass-ionomer restoration with claimed bioactivity. Moreover, the bio-inductive properties of the three tested materials are likely the same. Clinical significance: bioactive resin composite can be used as a retrograde filling.

## Introduction

Retrograde filling is indicated for teeth that cannot be treated with routine orthograde root canal therapy and history long-term orthograde RCT failures. Nowadays, several retrograde fillings are well-known such as mineral trioxide aggregate (MTA), zinc oxide eugenol and amalgam. The ideal material should possess certain criteria and thus selecting the most efficacious material is one of the essential prerequisites for obtaining successful treatment outcomes^1^. Proper retrograde filling should exhibit good bioactivity and biocompatibility. Also, it shows the characteristics of wet setting, easy manipulation, bonding to tooth substrate and radiopacity^2^.

A recently introduced category of an altered ion-releasing RMGI restorative materials has shown a promising results in the field of restorative dentistry^3,4^. Mineral Trioxide Aggregate is one of a calcium silicate-based biomaterial which was introduced in 1993 and since then it has been widely used by endodontists for the treatment of roots’ perforation, apexification as a root-end filling material^5^. It has been reported that MTA is considered as the gold standard for retrograde filling. This is attributed to its high biocompatibility, osteoinductivity, as well as, excellent chemical and physical properties. Conversely, this category of retro-grade filling suffered from certain limitations e.g. low-compressive strength, high alkalinity and relatively long-setting time^6^. To overcome the drawbacks of MTA, water-based bioceramic calcium silicate cements have been developed as a fully laboratory-synthesized biomaterials for root canal filling and repair applications, requesting an improvement in their clinical performance^7^. These materials showed a potent antibacterial activity, sealing ability and good biocompatibility. They are a convenient to utilize and supplied as ready-to-use paste in a white premixed putty form. The bioceramic putty absorbs water and moisture from the surrounding environment and initializes hydration of bi-calcium silicate and tri-calcium silicate to precipitate hydroxyapatite as an end product^8,9^.

This category of bioactive cements showed a promising result during grafting of freshly mixed ProRoot MTA implants in mandibular bony cavities of guinea pigs induced minimal inflammation with favorable healing response^10^. Also, it has been reported that ProRoot MTA when embedded in the mandibular symphysis of guinea-pigs showed declining toxicity over time with excellent biomineralizing and biological behavior^11^. A mild-to-moderate chronic inflammatory response with multinucleated and lymphocytes infiltration accompanied with formation of thin fibrous capsule/ dystrophic calcification could be observed one month after implantation of MTA Angelus in rat alveolar sockets, however this inflammatory response was gradually resolved within the following three months^12^. Likewise, in a rat femur model ProRoot MTA implantation showed better biological performance with lower inflammatory response and increased bone formation over longer follow up periods^13^. In addition, in a rabbit femur model, ProRoot MTA and an experimental dicalcium silicate cement showed well incorporation with the surrounding bone with diminished inflammatory response^14^.

Thus, the main purpose of the present study was to assess the intramedullary bone tissue reaction to an ion-releasing resin modified glass-ionomer restoration with claimed bioactivity (ACTIVA, MA, Pulpdent, USA) versus MTA-HP (Angelus PR, Brazil) and iRoot BP Plus (BioCeramix Inc., Vancouver, BC, Canada) as two calcium silicate-based cements of different properties in a rat model. This study was conducted to test the null hypotheses that that there were no significant difference between ion-releasing RMGI restoration and the tested 2 calcium silicate-based cements in intramedullary bone reaction and elemental structures. In performing this study, the guidelines of the Animal Research: Reporting In Vivo Experiments and the ARRIVE Checklist (https://www.nc3rs.org.uk/arrive-guidelines) were followed.

## Materials and methods

Materials used for the present study are listed in Table 1.

**Table 1:** Materials used in the present study.

Material	Manufacturer	Constituents	Manufacturer' instructions	Batch No
ACTIVA bioactive restorative	(Pulpdent, Watertown, MA, USA)	-Light curing (resin-based). Blend of diurethane and other methacrylates with modified polyacrylic acid (~ 53.2%), silica (∼ 3.0%), and sodium fluoride (∼ 0.9%)	-Two-paste system in auto mix syringe, has a triple cure reaction, light cure reaction, self-cure resin reaction and self-cure glass ionomer reaction -Light cure setting time (20 s)	VR2A1
Mineral trioxide aggregate-high plasticity (MTA HP)	(Angelus Co. Londrina, PR, Brazil)	-Powder: BI_2_O_3_, CaO, MgO, K_2_O, Na_2_O, Fe_2_O3, SO_3_, SiO_2_, Al_2_O_3_ -Liquid: distilled water with organic plasticizer	-Powder: Liquid mixing ratio 1:1 -Mixing tool: Stainless steel mixing spatula -Setting time (15 min)	43,505
iRoot BP Plus	(Innovative BioCeramix Inc. Canada)	-Paste containing: calcium silicates, zirconium oxide, tantalum pentoxide, calcium phosphate monobasic	-Ready-made paste applied directly -Setting time (2 h)	1807BPPS

### Sample size calculation

The sample size of this animal study was statistically determined using G* Power 3.1.9.2 software. The test family was F tests and the statistical test was ANOVA: fixed effects, omnibus, one-way. The type of power analysis was A priori: Compute required sample size-given α, power, and effect size. The input parameters were an error probability (α) of 0.05, an effect size (f) of 0.40, a power of 0.95 and number of groups was 4. The estimated sample size was 112. (28 rats/ group) and as the surgical defects were performed bilaterally (14 rats/ group); thus, the sample size was 56 rats.

### Animal selection and ethical approval

Fifty-six adult, male, pathogen-free rats (Rattus Norvegicus Albinus, Wistar), weighing approximately 180–200 g were obtained from Institute of Basic and Clinical Pharmacology Research Centre of Xiang Ya Medical school, Central South University. Rats were selected and housed in standard cages and used in accordance with the guide for care and use of laboratory animals and the surgical procedures were conformed according to the guidelines of ISO 10993-2 (Animal welfare requirements) and ISO 10993–1 (Part 6: tests for local effects after implantation)^15,16^. All experimental steps were performed after obtaining the approval from the ethical committee of Central South University for animal experiments (No: 20180025). The rats were housed at a temperature under 22 °C and at 65%-70% relative humidity. All rats were maintained in a 12-h light and 12-h dark cycle with normal dietary nutrition.

### Study design

The Research Randomizer software package (https://www.randomizer.org/) was used to randomly assign the rats into 4 equal groups (I, II, III and IV), 14 animals per each group. A surgical intramedullary bi-lateral tibial bone defects were performed in rats of the control group I (GI) and left without any treatment to be considered as controls (n = 28). The rats of groups II, III and IV were handled as group I except that the tibial bone defects were filled with with ACTIVA (Pulpdent, USA), MTA HP (Angelus High Plasticity, Londrina, PR Brazil), and iRoot BP Plus (Innovative BioCeramix Inc., Vancouver, BC, Canada), respectively.

### Surgical procedures

Anesthesia was performedafter intraperitoneal injection of ketamine hydrochloride and xylazine hydrochloride (3: 1 respectively; 0.05 mL/100 g of weight). Bilaterally, the fur leg was clipped, and operation site was wrapped in a sterile fashion with 0.12% chlorhexidine and the residual antiseptic was removed with sterile moistened gauze. A one cm longitudinal incision along the anteromedial border of tibia was performed using # 11 scalpel. The medial diaphyseal face of the tibia was exposed via blunt dissection using blunt scissor to expose a full mucoperiosteal flap with care taken during splitting the muscle to avoid injury to the tibial arterial and its venous return. A non-critical sized circular bony defect At of 2 mm diameter throughout the full cortical thickness and reaching the medullary space was performed using surgical round bur fitted on high-speed handpiece with copious irrigation of saline. Bony defects were filled manually with the tested materials according to the manufacture instructions using plastic spatula for the ion-releasing RMGI (ACTIVA) restorative material. Moreover, a stainless steel spatula was used for MTA HP and iRoot BP Plus cements, until extrusion occurred into the medullary space. Then, restorative material was light cured and cements were left to set. The bony defects in the control group were left to heal spontaneously without filling. The overlying muscles and skin were sutured with No. 0/3-resorbable suture.

Floxacillin antibiotic of 1 g and ketolac tromethamine analgesic of 10 mg were given for 4 days post surgery with a dose of 0.34 mg/kg. Regular daily checkup for the physical status and the healing course of the rats was performed after surgery. Postoperatively, after one month, each rat was euthanized by administering an anesthetic overdose with ketamine hydrochloride and xylazine hydrochloride (1: 1 respectively; 0.15 mL/100 g of weight; I/P) in accordance with ISO standards 10993-1 for prolonged-permanent long-term contact of implant devices for bone tissue. The areas of interest including bone defects were surgically retrieved and prepared for routine histological investigation, SEM examination and EDX elemental analysis. All the surgical procedures were performed by the author A.A and placement of the fillings was blinded in all restorative groups, except, ion-releasing RMGI (which has a unique delivery system).

### Histological specimen processing

The tibiae bones (n = 14/group) were fixed in 4% paraformaldehyde, decalcified in 10% EDTA (pH 7.4), longitudinally sectioned at the area of each bone defect. Decalcified tissues were processed in a tissue processor using ascending concentrations of ethanol, embedded in paraffin and sectioned at a thickness of 5 µm. After deparaffinization in xylene and rehydration in graded ethanol, hematoxylin and eosin (H&E) and Masson’s trichrome staining were performed according to Suvarna et al.^17^, technique. Slides were photographed using Leica Microscope (Leica Microsystems GmbH, Wetzlar, Germany) adapted with a DFC420 camera under × 10 magnification to observe the amount of new bone formation, inflammatory cell response, angiogenic process, granulation tissue formation, and then under × 40 magnification to semiquantitatively estimate the number of osteoblast and osteoclast cells. The histological scoring system was performed according to the criteria described by Abid et al.^18^, with slight modification (Table 2).The histological evaluation was performed blindly by 2 authors MG and WA. The inter- and intra-examiner reliability was verified by using Kappa test with mean percentage agreement of 90%.

**Table 2:** Parameters and scores used to test the healing effect of the tested materials.

Parameter	Score 0	Score 1	Score 2	Score 3
New bone formation	Absent	Present at the periphery	Present centrally	Present centrally and at the periphery
Inflammatory response	None	Mild	Moderate	High
Angiogenesis	Absent	2–4 vessel per site (mild)	4–6 vessel per site (moderate)	7–8 vessel per site (profound)
Granulation tissue	Absent	Present at the periphery	Present centrally	Present centrally and at the periphery
Osteoblasts	Absent	Present at the periphery	Present centrally	Present centrally and at the periphery
Osteoclasts	Absent	Present at the periphery	Present centrally	Present centrally and at the periphery

### Specimen processing and morphological analysis using SEM and EDX

The retrieved specimens (n = 14/group) were fixed in a solution containing 2.5% glutaraldehyde and 2% paraformaldehyde and then stored for 24 h overnight at 4 °C. The specimens were precisely cut into thin sections using ISOMET 4000, crossing the operation area and passing through the material, to expose the interface with surrounding bone for micromorphological surface analysis. The prepared disks were then washed three times for 15 min with phosphate buffered saline (pH 7.4) and fixed again in a solution of 1% sodium phosphate buffer osmium tetra oxide for 90 min. The exposed specimens’ surfaces were subjected to wet polishing using 600-, 800-, 1200-, 1600-, 1800- and 2000-grit SiC paper, cleaned using ultrasonic cleaner containing deionized water for 30 s. Finally, the specimens underwent graduate dehydration with ethanol. The specimens were dried and coated with gold (SPI Sputter coater, USA). Examination of the processed disks is performed using JEOL JSM 6510LV scanning electron microscope (Tokyo, Japan).

Energy dispersive X-ray analysis (EDX) for elemental microchemical determination was performed randomly for all specimens at the same magnification for the same areas with approximately fixed scanned area to evaluate the relative element content. EDX microanalysis weight was performed on bone material interface in selected frame to analyze entire areas and the elements were calculated from the data to evaluate the degree of mineralization of the newly formed bone. EDX element mapping was performed at the same area to detect the element distribution within the mature and newly formed bone and their presence in the surrounding tissues. Element mapping was performed through the bone-interface-material and were performed to detect the variation content of Ca, P, Si, Ta, Na, K, Al, Ba, Zr, C and O from the mature pre-existing bone towards the implanted material.

## Results

### Clinical findings

**Figure 1:**
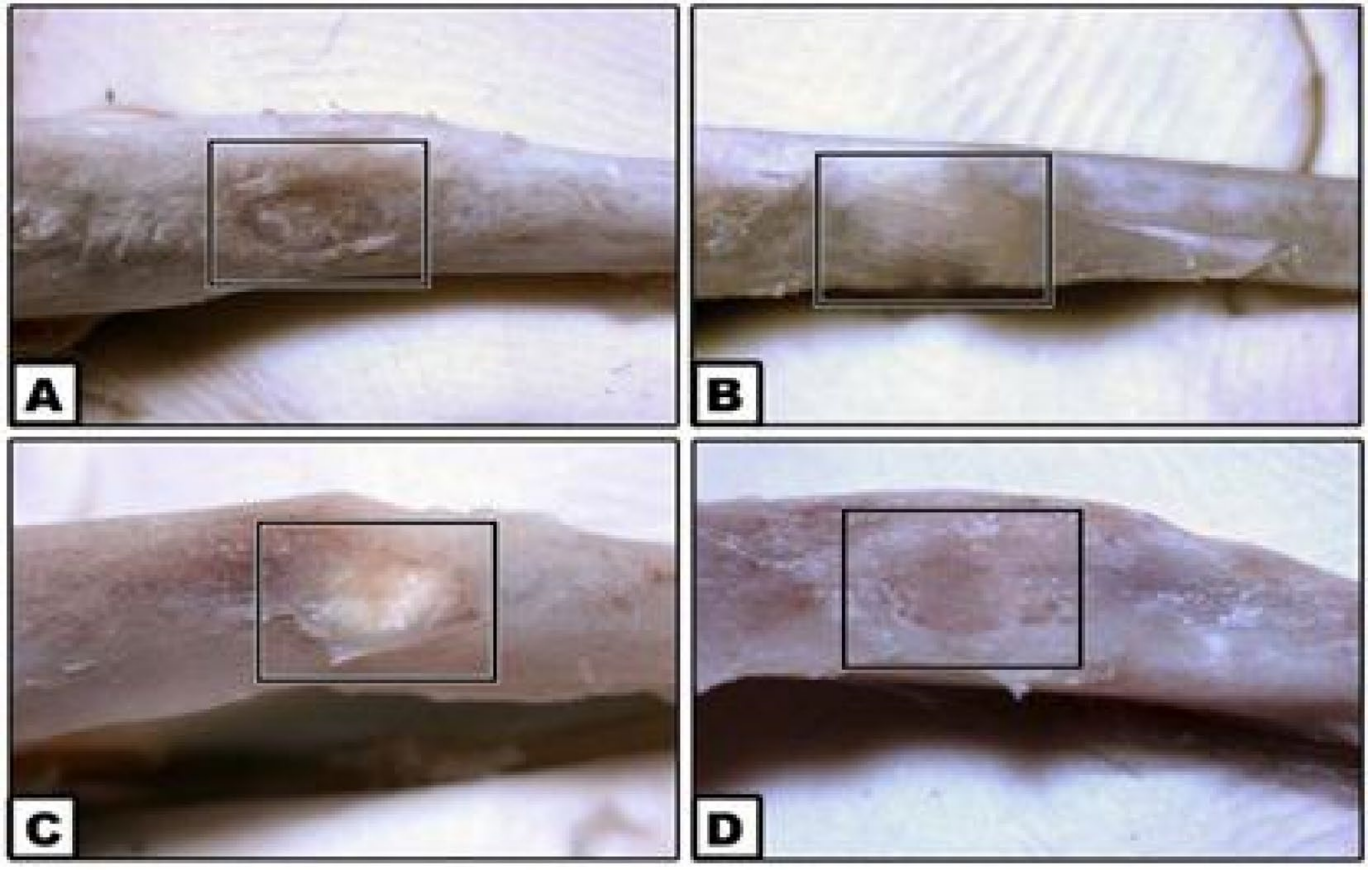
S9tereomicroscopic pictures showing healing of the surgical tibial bone defects after one-month. (**A**) Control, (**B**) ACTIVA, (**C**) MTA HP and (**D**) iRoot BP Plus.

Generally, recovery of rats was observed after 4 days post-surgery as they returned to their routine activities, such as walking, grooming and routine dietary habits. The rats showed normal chewing efficiency without any weight loss or postoperative complications. In addition, stereomicroscopic pictures for the tibial bone of all groups showed an acceptable healing in the control one, a defect filled with ACTIVA restorative material with better osteointegration with the old bone and an excellent healing was noticed in both MTA HP and iRoot BP Plus groups as they showed marked disappearance of bone defects and the boundaries of osteotomy gaps revealed better osteointegration with the old bone (Fig. 1).

### Histological results

The outcome of the Kolmogorov–Smirnov normality test showed that data did not follow the normal distribution and accordingly non-parametric statistical analysis was performed. Kruskal Wallis test revealed total significant differences between the control, ACTIVA, MTAHP and iRoot BP Plus groups (p < 0.05). Meanwhile, Mann–Whitney U test showed significant difference between control and ACTIVA groups, Control and MTA HP groups, control and iRoot BP Plus groups. ACTIVA and MTA HP groups, ACTIVA and iRoot BP Plus (p < 0.05) with no significant difference between MTA HP and iRoot BP Plus (p > 0.05).

**Figure 2:**
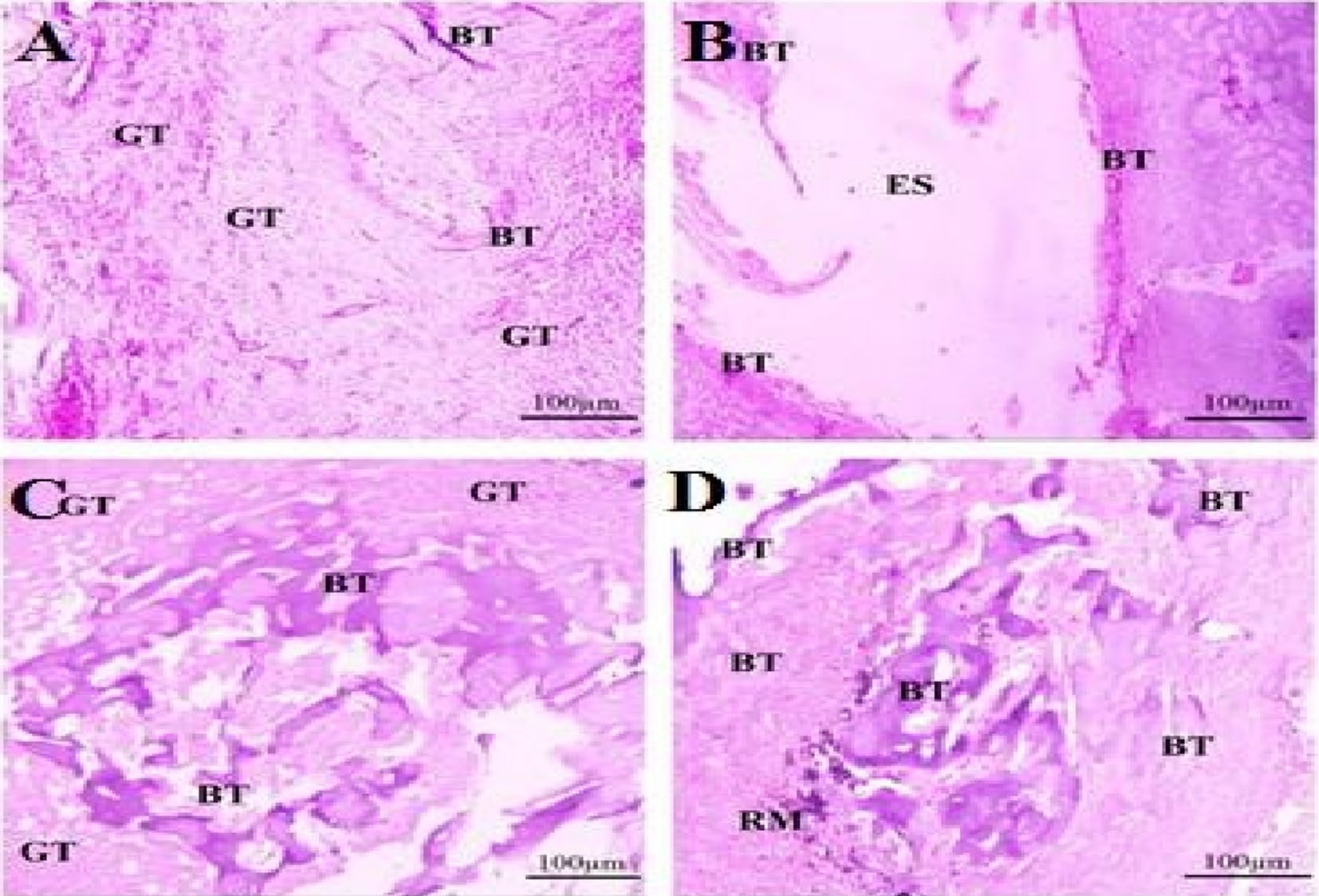
Photomicrograph of decalcified sections stained with H&E showing bone healing of tibial defects after one month. (**A**) The bone defects of the control group containing scanty thin trabeculae of woven bone (BT) with thick bands of typically organized granulation tissue (GT). (**B**) Bone defects of ACTIVA group showing an empty space (ES) that was previously occupied by the restorative material and it is surrounded by woven bone trabeculae (BT) at the interface with the medullary bone. (**C**) Bone defects of MTA HP group are partially filled with woven bone trabeculae (BT) with wide marrow spaces and a little amount of mature granulation tissue (GT) characterized by a decrease in the angiogenesis and an increase in collagen deposition and the bone defects are surrounded by woven bone trabeculae (BT) at the interface with the medullary bone. (**D**) Bone defects of iRoot BP Plus group showing marked woven bone formation of normal trabecular structures (BT) with narrow marrow spaces and a lesser amount of well-organized and mature granulation tissue formation (GT) with a residual material (RM) maintained in the granulation tissue (GT) and the bone defects are surrounded by woven bone trabeculae (BT) at the interface with the medullary bone, scale bar = 100 µm.

**Figure 3:**
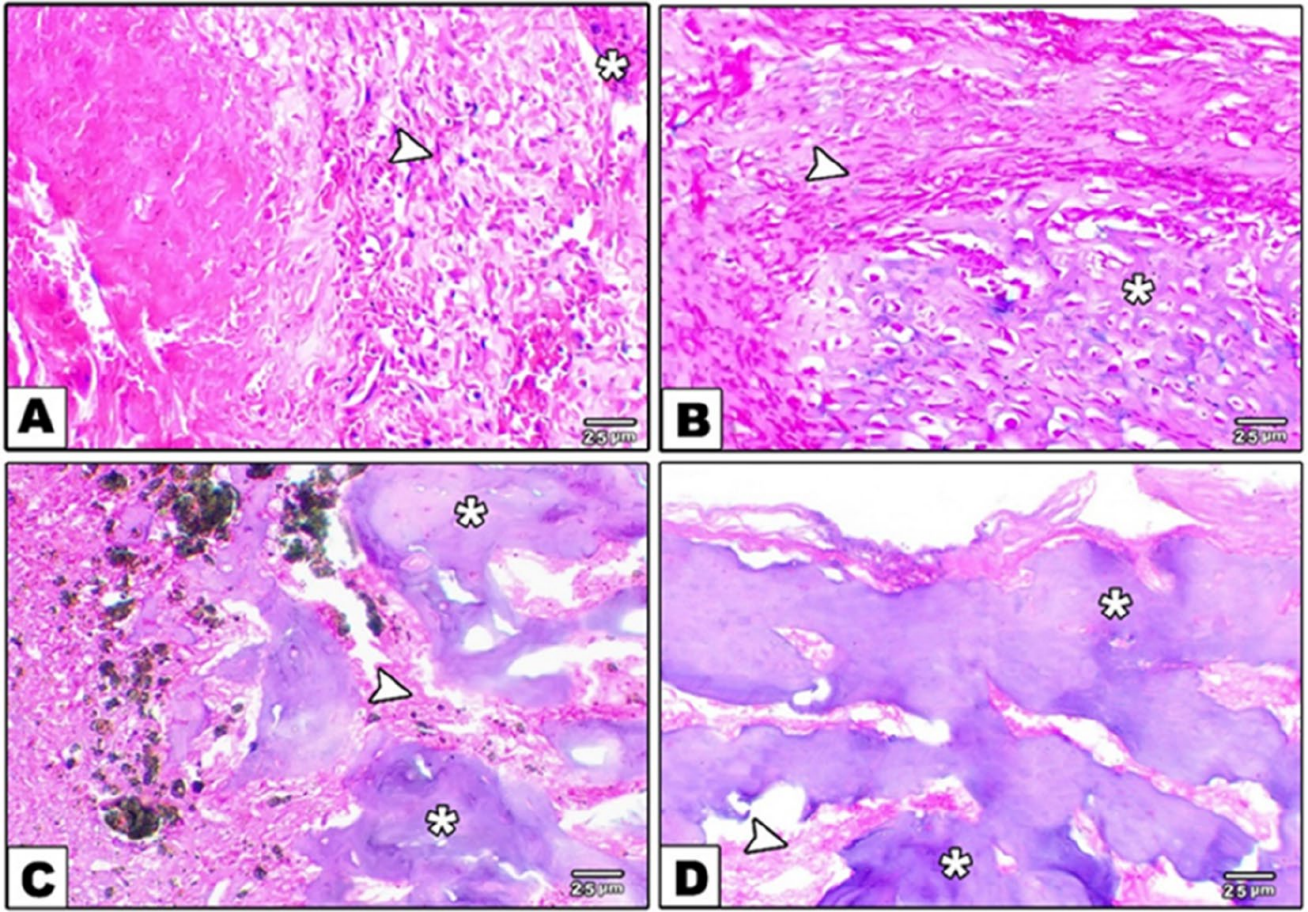
Photomicrograph of decalcified sections stained with H&E showing bone healing of tibial defect after one month. (**A**) The central part of the of the control bone defects containing thick bands of typically organized granulation tissue (arrowhead) and scanty thin trabeculae of woven bone (asterisks). (**B**) Bone defect of ACTIVA group showing woven bone trabeculae at the interface with the medullary bone (arrowhead) with osteoblastic cells proliferation within osteoid matrix (asterisks). (**C**) The central part of the bone defects of MTA-HP group showing woven bone trabeculae with wide marrow spaces (asterisks) and a little amount of mature granulation tissue (arrowhead). (**D**) The central part of the bone defects of iRoot Plus group showing marked woven bone formation of normal trabecular structures with narrow marrow spaces (asterisks) and a lesser amount of well-organized and mature granulation tissue formation (arrowhead), scale bar = 25 µm.

Control group sections showed scanty, very thin, new bone trabeculae of immature woven type located mostly at the peripheral part of the tibial bone defects (n = 25) with greater amount of thick bands of typically organized granulation tissue of tightly packed collagen bundles were located centrally and at the periphery (n = 24). The granulation tissue had moderate inflammatory cell response (n = 24)) and profound sprouting angiogenesis (n = 24). Osteoblastic activity was noticed only in the peripheral part of the defect area (n = 20) and less likely at the central one (n = 8) whereas osteoclastic activity was completely absent at both areas (Figs. 2A and 3A, Table 3). Meanwhile, bone defects of ACTIVA group showed an empty space that was previously occupied by the restorative material with thick, newly formed, immature woven bone trabeculae presented only at the marginal area (n = 28). The boundaries of the defect area demonstrated mild inflammatory cell response (n = 28), moderate sprouting angiogenesis (n = 23), absence of granulation tissue (n = 26) with marked osteoblastic and osteoclastic activities (n = 26) (Figs. 2B and 3B, Table 3).

**Table 3:** Results of scoring systems used to test the healing effects of the tested materials on tibial bone defects with the different parameters.

Groups	New bone formation				Inflammatory response				Angiogenesis				Granulation tissue				Osteoblasts				Osteoclasts			
	0	1	2	3	0	1	2	3	0	1	2	3	0	1	2	3	0	1	2	3	0	1	2	3
Control	3 0.11	25 0.89	0 0.0	0 0.0	0 0.0	0 0.0	24 0.86	4 0.14	0 0.0	1 0.04	3 0.11	24 0.86	0 0.0	0 0.0	4 0.14	24 0.86	0 0.0	20 0.71	0 0.0	8 0.29	28 0.100	0 0.0	0 0.0	0 0.0
ACTIVA	0 0.0%	28 0.100	0 0.0	0 0.0	0 0.0	28 0.100	0 0.0	0 0.0	0 0.0	5 0.18	23 0.82	0 0%	26 0.93	2 0.07	0 0.0	0 0.0	0 0.0	26 0.93	0 0.0	2 0.07	2 0.07	26 0.93	0 0.0	0 0.0
MTA HP	0 0.0	0 0.0	0 0.0	28 0.100	0 0.0	24 0.86	4 0.14	0 0.0	0 0.0	1 0.04	26 0.93	1 0.04	0 0.0	5 0.18	21 0.75	2 0.07	0 0.0	0 0.0	0 0.0	28 0.100	0 0.0	0 0.0	1 0.04	27 0.96
iRoot BP Plus	0 0.0	0 0.0	0 0.0	28 0.100	0 0%	26 0.93	2 0.07	0 0.0	0 0.0	4 0.14	24 0.86	0 0.0	0 0.0	9 0.32	18 0.64	1 0.04	0 0.0	0 0.0	0 0.0	28 0.100	0 0.0	0 0.0	2 0.07	26 0.93

Bone defects of MTA HP group were partially filled with thick and newly formed woven bone trabeculae with wide marrow spaces presented centrally and at the periphery (n = 28) with little amount of mature granulation tissue at the central part (n = 21). The granulation tissue was characterized by mild inflammatory cell response (n = 24), moderate sprouting angiogenesis (n = 26). Osteoblastic (n = 28) and osteoclastic (n = 27) activities were found at the central and peripheral areas (Figs. 2C and 3C, Table 3). While bone defects of iRoot BP Plus group showed marked woven bone formation of normal trabecular structures with narrow marrow spaces presented centrally and at the periphery (n = 28) and a lesser amount of well-organized and mature granulation tissue formation with a residual material maintained in the granulation tissue. The granulation tissue was characterized by mild inflammatory cell response (n = 26) and moderate sprouting angiogenesis (n = 24). Osteoblastic (n = 28) and osteoclastic (n = 26) activities were found at the central and peripheral areas (Figs. 2D and 3D, Table 3).

Regarding the parameters tested, Kruskal Wallis test revealed total significant differences between the control, ACTIVA, MTAHP and iRoot BP Plus groups (*P* = 0.000). Meanwhile, Mann–Whitney U test showed significant difference between control and ACTIVA groups, Control and MTA HP groups, control and iRoot BP Plus groups. ACTIVA and MTA HP groups, ACTIVA and iRoot BP Plus with no significant difference between MTA HP and iRoot BP Plus (Table 4).

**Table 4:** Results of the mean rank, Kruskal–Wallis and Mann–Whitney U statistical tests for the scoring systems used to test the healing effects of the tested materials on tibial bone defects with the different parameters.

Parameter	Control	ACTIVA	MTA HP	iRoot BP Plus	P^†^
New bone formation	26.11	32.89	83.50	83.50	0.000
		P1^‡^ = 0.026	P1^‡^ = 0.000, P2^‡^ = 0.000	P1^‡^ = 0.000, P2^‡^ = 0.000, P3^‡^ = 1.000	
Inflammatory response	95.93	39.50	47.21	43.36	0.000
		P1^‡^ = 0.000	P1^‡^ = 0.000, P2^‡^ = 0.040	P1^‡^ = 0.000, P2^‡^ = 0.015, P3^‡^ = 0.392	
Angiogenesis	91.23	41.73	49.75	43.29	0.000
		P1^‡^ = 0.000	P1^‡^ = 0.000, P2^‡^ = 0.054	P1^‡^ = 0.000, P2^‡^ = 0.018, P3^‡^ = 0.099	
Granulation tissue	94.00	15.00	61.23	55.77	0.000
		P1^‡^ = 0.000	P1^‡^ = 0.000, P2^‡^ = 0.000	P1^‡^ = 0.000, P2^‡^ = 0.000, P3^‡^ = 0.196	
Osteoblasts	39.50	27.50	79.50	79.50	0.000
		P1^‡^ = 0.038	P1^‡^ = 0.000, P2^‡^ = 0.000	P1^‡^ = 0.000, P2^‡^ = 0.000, P3^‡^ = 1.000	
Osteoclasts	15.50	41.50	85.00	84.00	0.000
		P1^‡^ = 0.000	P1^‡^ = 0.000, P2^‡^ = 0.000	P1^‡^ = 0.000, P2^‡^ = 0.000, P3^‡^ = 0.556	

^†^Kruskal Wallis, ^‡^Mann–Whitney U, P1: significance Vs control, P2: significance Vs ACTVA and P3: significant Vs MTA HP, Significant at ≤ 0.05.

### Micromorphological analysis results

**Figure 4:**
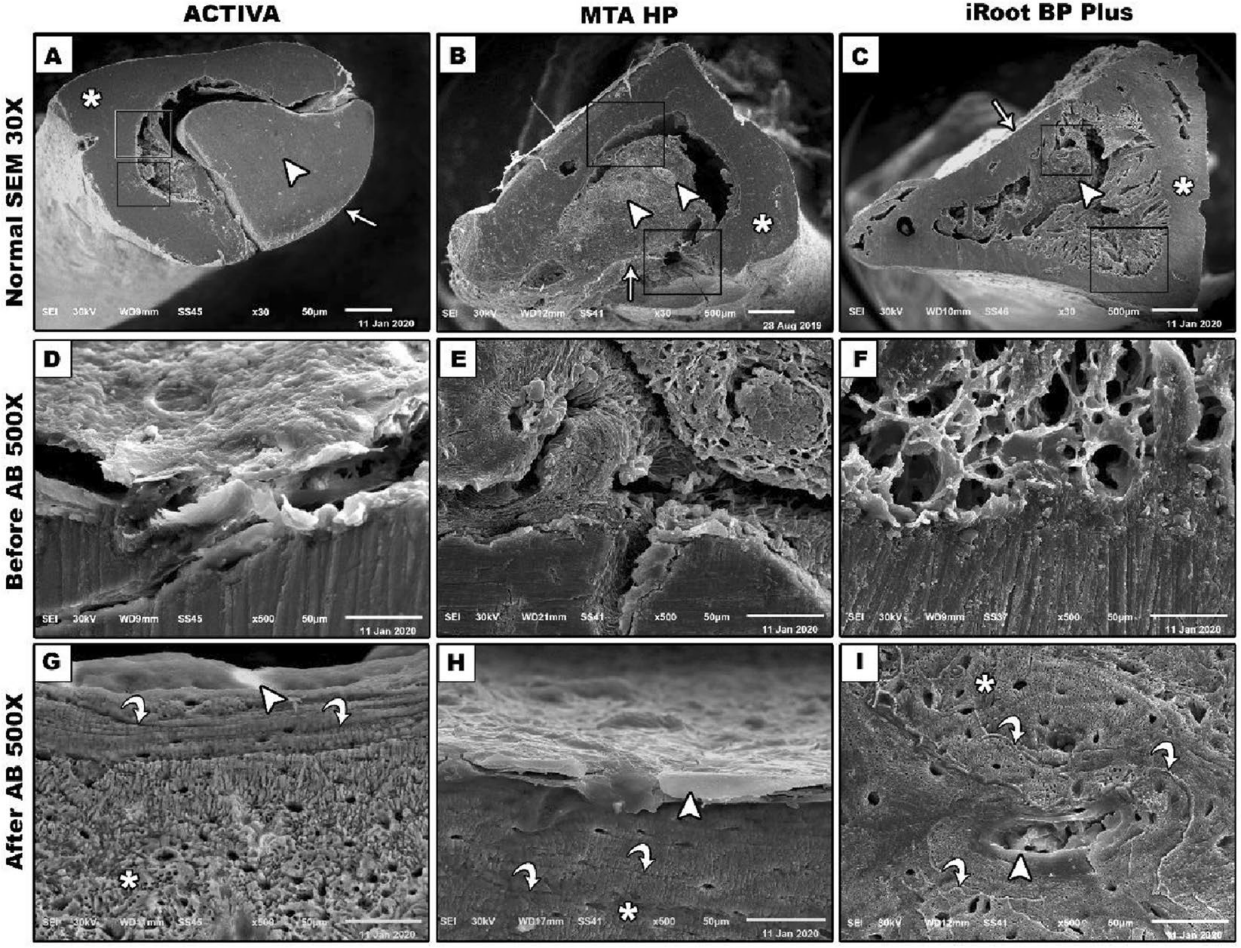
SEM morphological analysis of the materials-bone interface at 500 × magnification before and after acid–base challenge. ACTIVA showed signs of bioactivity with incremental deposition of the newly formed bone along the interface. The perfect contact with absence of gaps of the newly formed bone with the MTA HP and iRoot BP plus with material integration.

Bone is identified by the vascular canal and osteocyte lacunae. After acid base challenge, osteoblastic-osteocyte lacunae, osteocyte lacuna-canalicular network and osteonal lamellar pattern on the surface of trabecular bone was clearly observed. Newly formed woven bone has grown in close approximation to the underlying biomaterials. It appears less homogenously mineralized with the presence of non-remodeled islands of mineralized bone. The boundaries between secondary osteons and interstitial bone and between individual trabecular packets are formed by cement lines, which are relatively hyper mineralized in comparison and therefore appear brighter, forming the interface between the old bone and the new bone. Ion-releasing RMGI (ACTIVA) showed an intimate contact and signs of bioactivity with incremental deposition of the newly formed bone along the interface. The representative images showed also a perfect contact with absence of gaps between newly formed bone and the residuals of MTA HP and iRoot BP plus (Fig. 4).

### Elemental analysis outcome

The EDX results for the control sample representing the normal bone which known to be consists mainly of two compartments (Organic matter) represented by collagen and non-collagenous proteins resulting on the presence of both carbon (kα) and oxygen (kα) lines originally located at 0.2774 and 0.525 keV respectively.

**Figure 5:**
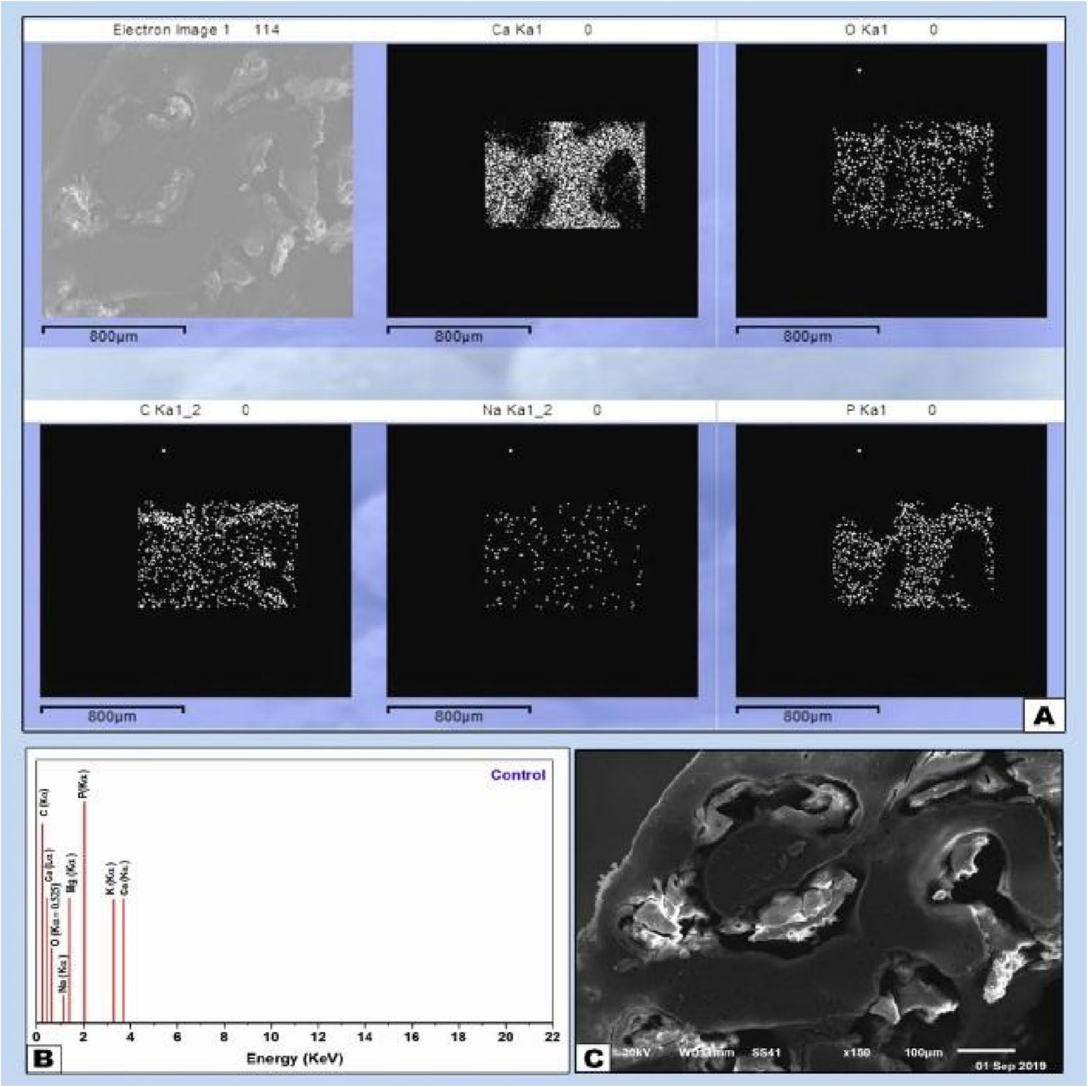
(**A**) EDX element mapping showing increased amount of C and O and decreased amount of Ca. (**B**) EDX analysis of control samples representing mineral amounts in the old bone towards the surgical site and the medullary space (**C**) SEM micrograph X150 of the control sample showing wide marrow spaces and irregular healing pattern.

**Figure 6:**
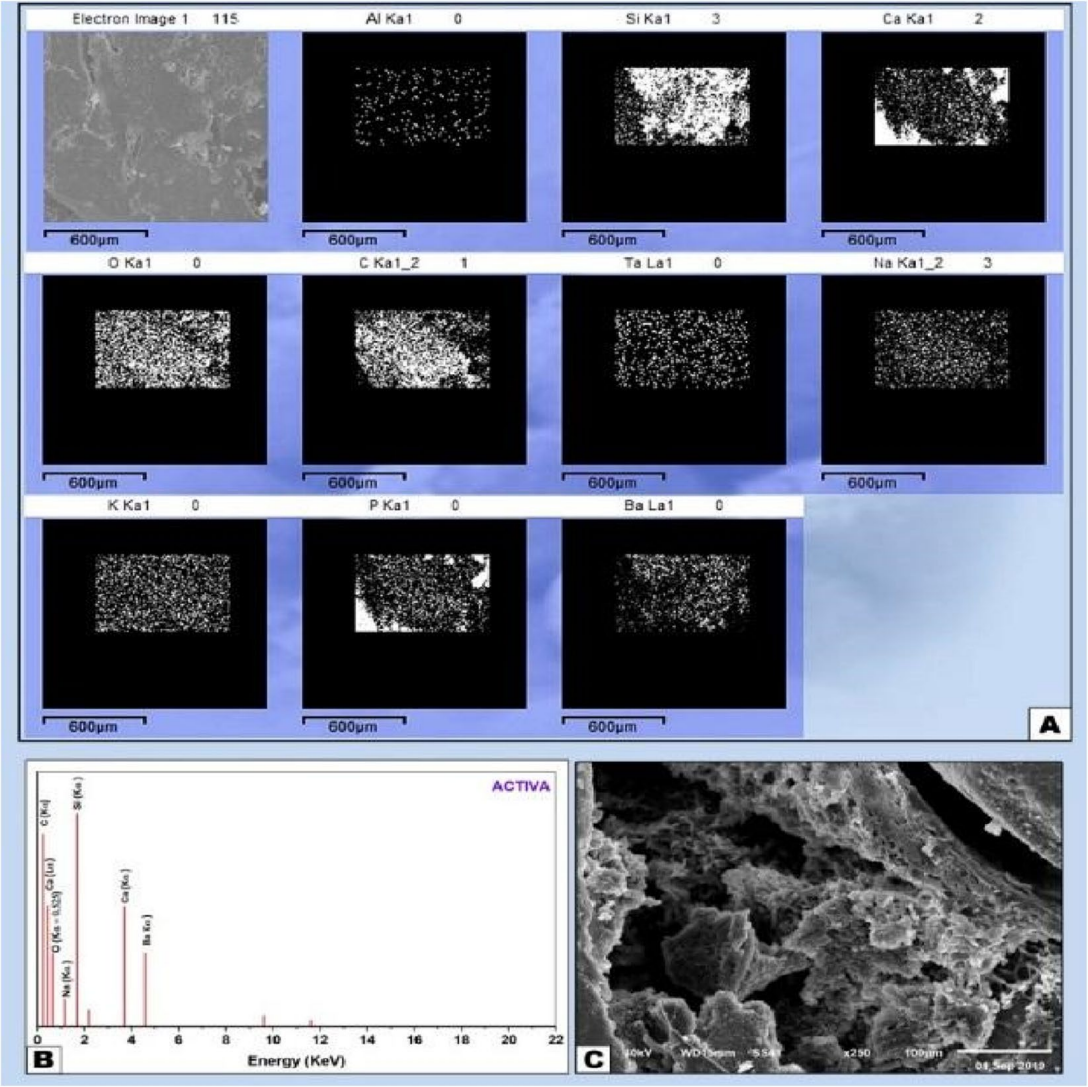
(**A**) EDX element mapping showing lower amount of Ca and P which indicate lower mineralization and increased amounts of Si from the material constituents. (**B**) EDX analysis of ACTIVA sample with the peaks representing the mineral amounts in the old bone towards the surgical site and the medullary space and the newly formed bone through the interface (**C**) SEM micrograph X250 showing healing of the ACTIVA sample with evidence of neo bone formation and irregular healing pattern.

**Figure 7:**
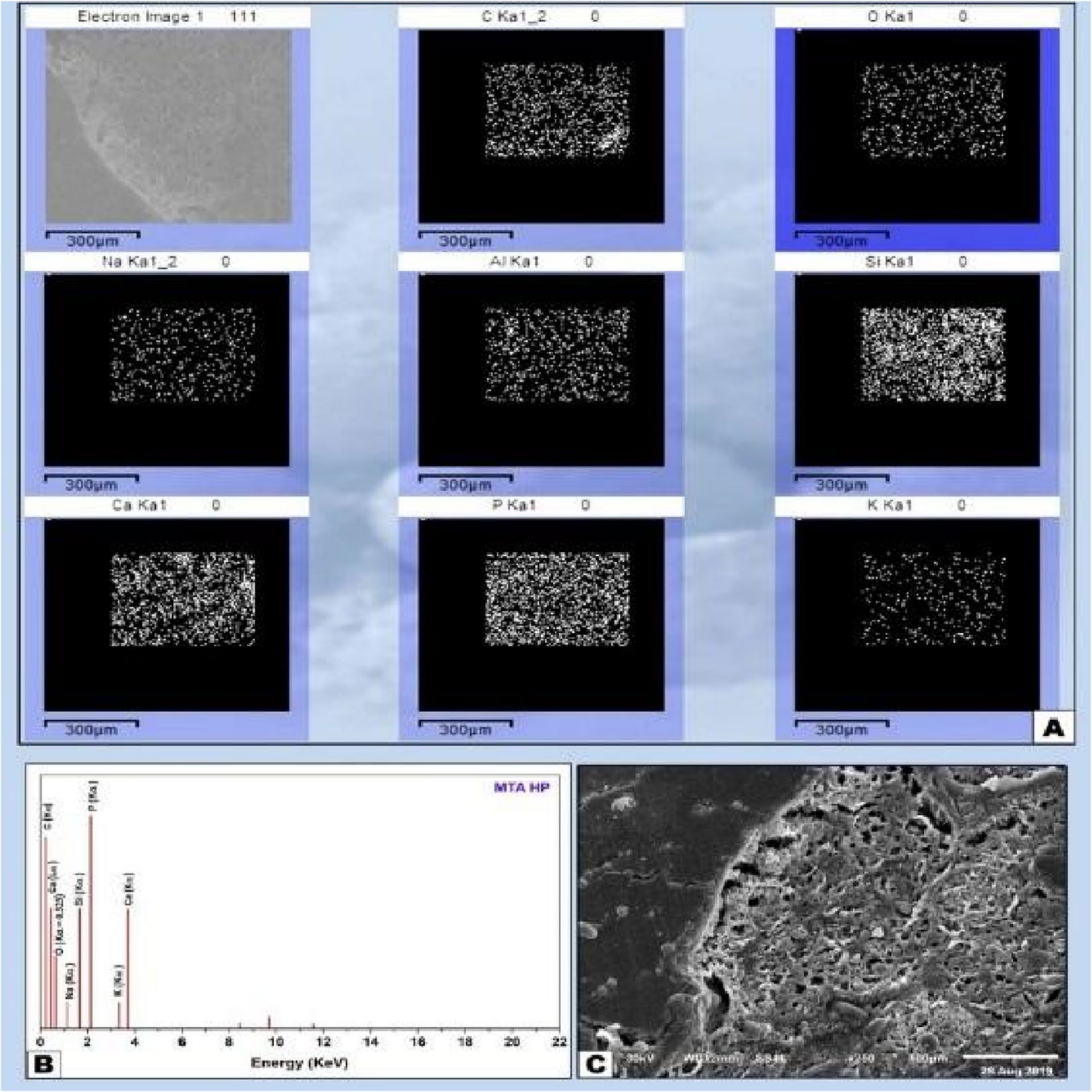
(**A**) EDX element mapping showing domination of Ca, Si and P which indicate good mineralization and presence scattered distribution of Al from the material constituents. (**B**) EDX analysis of MTA HP sample with the peaks representing the mineral amounts in the old bone towards the surgical site and the medullary space and the newly formed bone through the interface (**C**) SEM micrograph X250 showing healing of the MTA HP sample with narrow marrow spaces and new bone formation.

**Figure 8:**
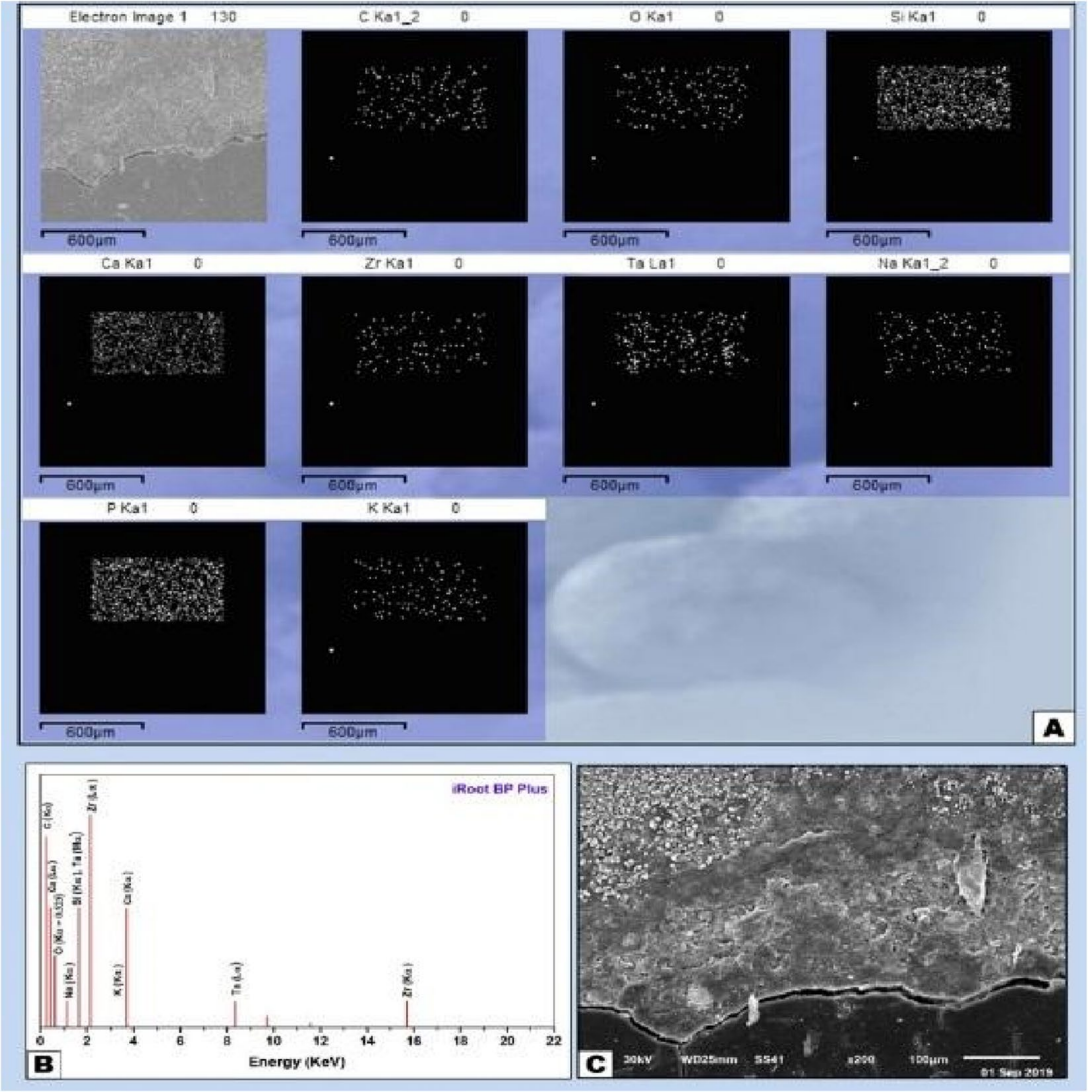
(**A**) EDX element mapping showing higher ratios of Ca, Si and P which indicate deeper mineralization and presence scattered distribution of Ta from the material constituents. (**B**) EDX analysis of iRoot BP plus sample with the peaks representing the mineral amounts in the old bone towards the surgical site and the medullary space (**C**) SEM micrograph X250 showing healing of the iRoot BP plus sample with neo bone formation with complete healing patte.

The lesions of the control group specimens were filled with recently created trabecular bone with limited marrow spaces. EDX tests (Ca and P analysis) indicated a lower degree of mineralization. Lower amounts of Ca and P was expressed in the mapping analysis corresponded by higher amounts of O and C with wider bone marrow spaces side (Fig. 5A–C). Figures 6, 7 and 8 represent the EDX analysis results for ACTIVA, MTA HP and iRoot BP Plus materials respectively. This indicated sharp lines assigned to the main compositions previously mentioned and their respective transitions and energies.

The histological segment observed by SEM showed the signs of bioactivity along the material-bone interface at the surgical defect with the formation of a layer of newly formed bone when Ion-releasing RMGI (ACTIVA) was put directly in contact with the sample on its medullar side (Fig. 6A–C). EDX and mapping analysis of the newly formed bone at the material-bone interface showed a lesser amount of Ca and P and increased amounts of C and O representing the organic components, indicating the lower degree mineralizing ability of ACTIVA in comparison with iRoot BP plus and MTA-HP in terms of bioactivity. Interestingly a noticeable increased amount of silica was noticed at the interface between the material and inner medullary side which may represented as a constituent of the material. However, deposition of a newly formed bone was evident. In the direction from old pre-existing bone towards the material there was a sudden drop in the levels of distribution Ca and P inorganic elements which represents the decreased amount of mineralization of the newly formed bone at the interface.

The histological segment observed by SEM showed the surgical defect entirely filled with the material and newly created bone filling the medullary space and showed the osteoinductive properties of MTA-HP strictly in contact with the sample on its medullar side. EDX microchemical analysis shows its constituent elements, i.e., Ca, Si, Mg, Al with Bi residues and high amount of P in the samples (Fig. 7A–C). Ca and P revealed a pre-existing trabecular bone. Ca, P, lack of Bi, and traces of Si originating from the material were revealed by newly formed bone. In the pre-existing old bone, the Ca and P proportions were notably higher than in the newly developed bone, in particular in new trabeculae adjacent to the material (probably the newest bone). The high-magnification interface analysis at 250 × showed the complete interaction and integration of the newly formed trabeculae with the material and the absence of spaces was well demonstrated. In newly formed bones, no Bi or Al was found, but only at the interface.

The histological segment observed by SEM showed the surgical defect entirely filled with complete integration between material and newly created bone filling the entire medullary space and showed the superior bioactive properties of iRoot BP plus directly in contact with the sample on its medullar side (Fig. 8A–C). Its constitutive elements, i.e., Ca, Si, Zr, and traces of Ta, were seen on the material surface by EDX. Higher Ca and P material, traces of Si and lack of Na were shown by pre-existing trabecular bones. In newly formed bone intermingled with the material residues showed higher amount of Ca and P and decreased amounts of C and O which represents the organic components, indicating the higher mineralizing ability of iRoot BP plus in comparison to MTA-HP which is expressed by the mapping analysis. There was no interrupted continuity along the material-bone interface with total filling of the marrow spaces with newly formed trabecular bone intervened by the residue of iRoot BP plus material which is replaced by the newly formed bone represented by presence of small amounts of Ta.

## Discussion

In the current study calcium silicate cements used for endodontic retrograde filling were tested against newly Ion-releasing RMGI (ACTIVA) restorative material in rat model through intramedullary bone tissue reaction by using H&E histological analysis, SEM morphological and EDX elemental analysis for their bio-functionality and osteo-inductive characteristics in a trial to mimic oral conditions.

The old perception of biocompatibility was related to the disappearance of adverse tissue reaction to the tested material^19,20^. However, some materials were not found to induce any noteworthy interaction with the host living tissues^21^. These reactions which help the improvement of wound healing and granulation tissue formation. The recent description of biocompatibility is the ability of dental biomaterials to elicit favorable and beneficial reaction with oral tissues during its intended clinical usage^16,22^.

It has been found that the biocompatibility assessments produced by cell culture assays is not necessarily to be in agreement with animal in vivo biocompatibility implantation test^23^. Therefore, using animal models, like rodents and rabbits are suitable choice for testing biological, regenerative and mineralizing characteristics of new materials in less invasive and non-critical sized surgical procedures^24^.

The ingredients of the new ion-releasing resin composite restorative material contain glass particles and a hydrophilic ionic resin matrix that facilitates the diffusion of fluoride, phosphate and calcium ions into the surrounding environment^3,22,25^. It was reported that complete healing occurred following retrograde resin placement with 97% success rate^26^. Retrograde filling with composite exhibited complete healing in 74% of cases in correlation to 59% of cases treated with amalgam^19,22^. These materials showed a higher degree of biocompatibility when subcutaneously implanted in comparison to the calcium silicate-based cements as it decrease the degree of inflammation with a better healing potentiality^16^.

A combination of *in-vitro*, *ex-vivo*, and *in-vivo* laboratory testing are essential to reveal the biological properties of the structure and composition of implanted materials. *In-vivo* animal models give initial perceptions about the physiological status, biomechanical interaction and the hormonal response against a newly tested materials, starting from rodents and rabbits as small models and ending by dogs and pigs as large ones^27^. Rabbits and rodent animal models are the key source for studying the biological behavior and regenerative potential in implant studies^28^. They are excellent candidate for modeling bone infections and extra oral surgical procedures to test the osteoinductive potential of allogeneic and xenogeneic bioactive materials^22,28^.

The outcome of this study showed the osteoinductive ability of MTA HP, iRoot BP and ACTIVA with biomineralization and new bone formation around the materials which filled the medullary space of rat tibia. Moreover, inflammation with subsequent necrosis of the cortical bone in contact with the materials was absent. In the analysis process SEM and EDX were adopted as accredited methods for the assessment of the surface morphology, the type and the amount of mineral content of the bone-material interface^29^.

Specimen analysis showed strong co-relation between calcium and phosphorus (Ca/P) like stoichiometric hydroxyapatite. Zones of mineralization and bone-material interfacial mapping were marked by SEM imaging and EDX mapping; therefore, the differentiation between the old per-existing bone and newly formed bone and cortical and cancellous bone is explained by the differences in minerals constituents expressed by the EDX elemental analysis.

The areas of remodeling process and newly formed bone are expressed by the decreased amount of Ca and P ratios in some certain trabecular segments, while, increased amount of Ca and P with the decrease of C, Si and O indicates the directional profile from mineralizing interface towards the old mature bone.

EDS, EDX, EDAX, or EDXA simply known as Energy-dispersive X-ray spectroscopy representing a direct and powerful tool that enables scientists to analyze and identifying chemical composition of studied samples depending on the core electron ejection with high energy X-ray radiation (Moseley's Law) gives a direct correlation between frequency of emitted radiation and the atomic number of the atom^30^.

The data generated by EDX analysis consist of spectra showing peaks corresponding to the elements making up the true composition of the sample being analyzed. In bone, the most frequent application of EDX is measurement of Ca and P content in the extracellular matrix with the calculation of Ca/ P ratio. Local nanomechanical properties have been shown to correlate with Ca levels. Detection of Ca and P along the bone-biomaterial interface confirms the formation of new bone in direct contact with the biomaterial surface. EDX-mapping was performed, because this characterization method is considered an accurate method for detecting elemental distribution among the observation specimen.

Histological examination showed that intramedullary mediated bone new formation, osteoblastic differentiation and angiogenic proliferation were implanted by MTA HP, iRoot BP and ACTIVA. Both MTA HP and iRoot BP materials showed strong biocompatibility, as only mild or moderate inflammatory infiltrate was evident quite close to the interface. While ACTIVA showed the least mineralization ability. This might be attributed to its high resinous content which countered its claimed bioactivity.

The study demonstrated the ability of an Ion-releasing RMGI (ACTIVA) and hydraulic calcium silicate cements to form osteoid matrix with activation of osteoblasts and achieving a direct bond between the materials and the surrounding bone matrix. The formation of favorable environment by calcium silicate cements for osteoblastic differentiation with new bone formation through interfacial active biological ions exchange and its effect on cellular activity could explicate the (bio-mineralizing) response of the tested materials. Bioactivity of a material is related to its chemical reactivity with living tissues through favorable interfacial ion exchange and precipitation reactions^31^, and for biomineralization by induction for osteogenic extra-cellular signaling with summoning cascades of cellular differentiation into osteoblasts with osteoid matrix lay down at the surgical site^32^.

Calcium silicate cements shown in a previous studies to have the ability to provide high alkaline pH in the media and release calcium ions after formation of hydrated silica-rich layer^33^. Therefore, generating a favorable environment at the interface with continuous provision of biologically active ions through the surface for apatite deposition^34^. In addition, silica Si ions as a byproduct has a role in new bone formation as it encourages the angiogenesis through elevating gene expression which is synergetic with the differentiation of osteoblasts and extracellular matrix synthesis and deposition^35^.

The setting mechanism of calcium silicate cements starts after hydration by water during the mixing process. The formed calcium silicate hydrate by-products formed after mixing have many phases as reported by Gandolfi et al^36^. These stages including porous colloidal CSH gel and radial acicular CSH crystals, rhombohedral crystals of portlandite, needlelike crystals of ettringite which is a hexacalcium alumina-tetrisulphate hydrate, and calcium mono- sulfoaluminate or calcium mono-carboaluminate. Moisture plays a major role in initiation of apatite formation (bioactivity) and nucleation process of calcium ions on the surface of hydrated calcium silicate particles, it goes through defined stages starting from rapid dissolution of calcium ions after the solid–liquid interface formed on the cement particles forming portlandite. A porous, fine-grained/fibrous and disorganized hydrated silicate gel layer is formed by alkaline etching; containing Si–OH silanol groups and negative surface charges that may act as nucleation sites for apatite formation. Portlandite crystals nucleate inside the hydrating wet paste due to a strong continuous outflux of calcium hydroxide from CSH that occurs during the first hours after mixing. Calcium phosphate and apatite deposit on cement surface as result of exposure to tissue fluids after being placed in vivo^37^. Calcium hydroxide formed reacts with carbon dioxide in the tissues to form calcified nodules which acted as nucleus for mineralization^38^. In addition, Zamparini et al.^39^ revealed that ready-made paste bioceramic materials containing tantalum pentoxide as radiopacifier showed higher calcium and silicate ions release.

The hydrophobic nature of resin component in light-cured calcium silicate containing dental materials make them less soluble than MTA. The pores found in the resin matrix which allow for water penetration with inward outward flux, therefore it could explain their capability of releasing of calcium through solubilization of calcium ions and calcium silicate hydrate and portlandite formation^40^. Morrow et al.^41^ compared the calcium releasing ability of ACTIVA, Dycal and TheraCal in 7 days period, results showed that ACTIVA released an amount of 0.72 μg/mm^2^ which was less than that released from Dycal (4.88 μg/mm^2^) and TheraCal 9.12 μg/mm^2^^41^.

In the context, it has been reported that hydroxy apatite particles formed on the surface of ACTIVA after 30 days of immersion in phosphate buffered saline after utilization of SEM, PulseTor SDD (Silicon Drift Detector) and Energy-dispersive X-ray spectroscopy (EDS) analysis^42^. It has been also shown that ACTIVA has the ability to form dentinal tubules integrated resin tags with 2.0–2.5 Ca/P ratio across the hybrid layer^3,42^.

Actually, in the present study ACTIVA showed promising biomineralizing response but the tested calcium silicate cements showed better performance in biomineralization, angiogenic activity with neovascularization and formation of capillaries close to the materials. In addition, MTA HP and iRoot BP were incorporated with surrounding bone showing intervening between old and newly formed bone without recognition of the materials exhibiting no interposed connective tissue, resorption lacunae inflammatory response, rejection or necrosis in the adjacent pre-existing bone and medullary side. No osteoclastic activity or multinucleated giant cells observed in the histologic analysis except for a few monocytes and lymphocytes existed. It can be assumed that the high alkaline pH played a significant role in inactivation of osteoclasts as explained in previous studies^43,44^, and previous study demonstrated the high alkaline pH of iRoot BP and MTA HP and nearly neutral pH for ACTIVA^45^.

In light of this study outcome, the null hypothesis was rejected. A part of the laboratory work of the study was performed during Covid-19 pandemic time and our research team faced some delay and shifting from the original timeframe due research laboratory closure. Also, further advanced radiographical methods (such as Micro-CT) is recommended in future studies.

## Conclusion

Calcium silicate-based cements (MTA HP & iRoot BP Plus) and resin-modified glass ionomer restorative material (ACTIVA) exhibit the ability to induce new bone formation through osteo-inductivity. The tested resin-modified glass ionomer restorative material does not induce fibrous encapsulation or any foreign tissue reaction with the direct contact between material superficial layer and mineralized bone matrix.

## Supplementary Information

Supplementary Information.

## Data Availability

All datasets generated or analysed during this study are included in this published article [and its Supplementary Information files]. Regarding the qualitative analysis (Histological and SEM micrographs), the authors already included the outcome of representative specimens from each group in the manuscript figures and tables, while the whole set of micrographs are available from the corresponding author on reasonable request.
